# Morphological, Biochemical, and Cytological Analyses of Deep-Sowing Tolerance in Sorghum Seeds

**DOI:** 10.3390/plants14091366

**Published:** 2025-04-30

**Authors:** Yutao Huang, Zhaotong You, Heyun Chen, Xiuhui Liu, Gaofu Mei, Heqin Liu, Dongdong Cao, Xueqiang Zheng, Guihua Zou

**Affiliations:** 1 Institute of Crop and Nuclear Technology Utilization, Zhejiang Academy of Agricultural Sciences, Hangzhou 310021, China; huangyutao@zaas.ac.cn (Y.H.); zhengxueqiang@zaas.ac.cn (X.Z.); 2 Institute of Virology and Biotechnology, Zhejiang Academy of Agricultural Sciences, Hangzhou 310021, China

**Keywords:** sorghum, deep-sowing emergence, mesocotyl elongation, phytohormones, glycometabolism

## Abstract

Deep-sowing tolerance (DST) is a key trait for the field germination of sorghum (*Sorghum bicolor* L.) seeds, especially in arid and semi-arid regions. However, the mechanisms of DST are poorly understood in sorghum. In this study, we compared two sorghum lines with contrasting tolerance to deep sowing for morphological, biochemical, and cytological changes during germination from deep soil (15 cm). The deep-sowing-tolerant (DT) line (Daluochui) showed 79% seedlings establishment (SE), while the deep-sowing-sensitive (DS) line (Xiaobailiang) showed no established seedlings at 7 days after sowing. Mesocotyl elongation is a key morphological change that accounted for the difference in seedling establishment between DT and DS. The mesocotyl elongation in DT was jointly established by both cell division and expansion. The levels of ethylene, auxin, and spermidine were markedly higher in DT than DS and were also supported by enzyme activity and qPCR, indicating that phytohormones play an important role in seed emergence from deep soil. Furthermore, α-amylose activity, soluble sugar, and ATP contents in DT were markedly higher than in DS, suggesting that there was a better energy supply in DT during deep-sowing emergence. The activities of endo-1,4-β-xylanase and endo-β-mannanase, as well as the expression of the corresponding genes, were higher in DT than DS. This study identified potential key regulatory factors that may control sorghum DST and yield potential, thus, providing new insights into the molecular mechanism of sorghum DST.

## 1. Introduction

Sorghum (*Sorghum bicolor* L.), as one of the most ancient cultivated crops, is widely grown in the world and has multiple applications in food, wine-making, feed, and silage [1,2]. The sowing depth of sorghum seeds affects germination speed and rate. Sorghum is generally sown at a depth of 3–5 cm in agricultural production. Deep (>7 cm) or shallow (no soil coverage) sowing can significantly reduce the germination rate of sorghum [3,4]. In the case of deep seeding, the thick overlaying soil increases the resistance to seed emergence and induces a higher risk of loss of seed vitality due to insufficient soil oxygen [5,6]. wNonetheless, in arid regions, the water shortage during the seed germination process is the major cause of the low emergence rate. Proper deep sowing contributes to increased root density of crops in the deep-soil-enhanced drought and lodging resistance and improved crop yield in arid and semi-arid regions [7,8,9]. Therefore, research on the molecular mechanisms underlying deep-seeding and soil emergence in sorghum seeds holds significant importance for sorghum production in arid regions.

The emergence of cereal crops (including corn, sorghum, and rice) is mainly achieved through coleoptile and mesocotyl elongation to push out of the soil layer [10,11]. Several studies demonstrated that mesocotyl elongation of corn and rice can improve the emergence rate under deep-sowing conditions and that the mesocotyl length is associated with the emergence speed and emergence rate [9,12,13]. Similar to corn and rice, the sorghum embryonic axis can be divided into epicotyl, mesocotyl, and hypocotyl; however, the sorghum epicotyl is extremely short, and the hypocotyl is not obvious. During the sorghum seed germination process, the mesocotyl elongation sends the seedling to the soil surface. Previous research reported that mesocotyl elongation is an important factor in determining the emergence rate under the deep-sowing condition in sorghum [4]. However, the molecular mechanism of sorghum mesocotyl elongation is rarely explored.

Studies on mesocotyl elongation in rice, maize, and Arabidopsis have revealed the important role of phytohormones in the process of deep sowing of seeds [14,15,16,17]. In *Arabidopsis thaliana*, gibberellin (GA) plays a core role in regulating the mesocotyl elongation, and the GA-DELLA complex can interact with other plant hormones, including jasmonic acid (JA), brassinosteroid (BR), and auxin (IAA), and affect mesocotyl elongation through regulating the downstream PIF3/PIF4 and cell wall remodeling [16]. Jiang et al. (2017) reported that phytohormones, including exogenous GA, abscisic acid (ABA), cytokinin (CTK), strigolactone (SL), indole-3-acetic acid, BR, and ethylene (ETH) can affect the mesocotyl elongation of rice [18]. Hu et al. (2014) found that SL and CK exerted antagonistic effects on regulating the mesocotyl elongation of rice [19]. Under the deep seeding condition, the endogenous hormones, including IAA, GA, CK, ABA, ETH, BR, and SL, accumulated in the mesocotyl of corn, regulating corn cell elongation and division [17]. However, the role of phytohormones in the deep-sowing tolerance and mesocotyl elongation of sorghum seeds has not been reported.

In plants, mesocotyl elongation is determined by cell growth, division, and morphogenesis [20]. The microtubule is a major component of the cytoskeleton, which exerts an essential effect on cell growth, division, and morphogenesis [21]. *OsP60-1* and *OsP60-2* promote the depolymerization of microtubules and increase GA content, thus promoting mesocotyl elongation of rice [22]. Moreover, mesocotyl elongation in the early stage was mainly determined by cell division and proliferation, while that in the middle and late stages, by rapid cell division and the subsequent increase in cell length [23]. The cell wall plays a key role in cell division, elongation, and morphogenesis, and the weakened cell wall aids mesocotyl elongation [7]. Cellulose is the major component of the cell wall, and the presence of a large number of hydrogen bonds between cellulose molecules renders a highly compact and stable cellulose microfibril (CMF) [24]. CMF is a major restricting factor for cell elongation. The enzymes responsible for degrading CMF in the cell wall of mesocotyl cells mainly include β-1,4-xylanase, β-xylosidase, and mannase, which are important for weakening the cell wall [25].

Starch represents a major storage polysaccharide in sorghum seeds [26]. Starch, which can degrade into soluble sugars, has an important effect on promoting mesocotyl elongation and seed germination. There are two enzymic routes related to starch degradation in sorghum, namely, amylase-catalyzed hydrolysis and starch phosphorylase-catalyzed phospholysis. According to Wang et al. (2022), hydrolysis, but not phosphorlysis, is the major cereal starch degradation process in rice seed germination [27]. Zhao and Wang (2001) found that α-amylase was important for starch hydrolysis in the seed germination of maize and rice [28]. The α-amylase level within germinating cereal grains has been suggested to be under the regulation of phytohormone. Based on Kim et al. (2006), gibberellic acid (GA) enhanced de novo α-amylase synthesis in aleurone layer cells [29].

## 2. Lines and Methods

### 2.1. Lines

From 110 sorghum lines collected in our laboratory, two sorghum lines with a significant difference in deep-sowing tolerance (DST) were selected according to the deep-sowing emergence test (sowing depth 15 cm), namely, the deep-sowing-tolerant (DT) line (Daluochui) and the deep-sowing-sensitive (DS) line (Xiaobailiang) [30]. The sorghum (*Sorghum bicolor* L. *Moench*) seeds were produced at the experimental farm of Zhejiang Academy of Agricultural Science (Hangzhou, China).

### 2.2. Deep-Sowing Emergence Test

Sorghum seeds with 14.5% moisture content were utilized for the deep-sowing emergence test (n = 50 for every replicate, 4 replicates). Seed disinfection was carried out with 0.1% NaClO solution for 15 min, followed by sowing in soil at 15 cm under a 12 h/12 h cycle and 25 °C conditions. Sorghum seeds sowed in soil at 5 cm depth served as a “control”. The seedling emergence was recorded at 5 and 7 days of germination. The mesocotyl and coleoptile length were determined at 7 days of germination.

### 2.3. Phytohormone Detection

GA and ABA were extracted from sorghum mesocotyls, as described by Huang et al. (2017) [31]. Specifically, GA and ABA contents in extracting solutions were analyzed with an HPLC system by utilizing the ultraviolet detector and the reversed-phase column (C18, 6.0 mm × 120 mm, particle size 5 mm; Shim-Pack CLCODS). Simultaneously, the mobile phase, including methanol/water (64:36, *v*/*v*), was adopted with a flow rate of 11 mL·min^−1^. ABA and GA level detections were performed with four biological replicates.

Ten sorghum seeds or seedlings were enclosed in a 100 mL air-tight container under 25 °C for a 3 h period to analyze ethylene generation (nmol·g^−1^· h^−1^·FW). Later, 1 mL headspace gas was sampled and injected in the gas chromatograph (model Agilent, 6890 N, USA) equipped with an activated alumina column and flame ionization detector. The parameters below were adopted for measurement: chromatograph column, 30 m capillary alumina column (Agilent 19091 J-413), HP-55% phenyl methyl siloxane; detector and column temperatures at 150 °C and 80 °C, separately; and the 40 mL· min^−1^ (carrier gas) flow rate. Ethylene generation was analyzed by four biological replicates.

Total CK and indole-3-acetic acid contents in sorghum mesocotyls were measured through high-performance liquid chromatography–electrospray ionization–tandem mass spectrometry (HPLC–ESI–MS/MS) [32,33]. The QTrap5500 triple quadrupole mass spectrometer (Sciex Applied Biosystems) and the Shimadzu LC10ADvp (Shimadzu) were utilized to determine total CK and IAA contents within extracted samples. Typically, 20 µL sample aliquots were added to the reverse phase C18 column (Kinetex 2.6 u C18 100 A, 2.1 × 50 mm; Phenomenex). HPLC–ESI–MS/MS data were processed by Analyst (v. 1.6.2) software (Sciex Applied Biosystems) for analyzing peak area based on isotope dilution analysis. Total CK and IAA contents in sorghum line-derived samples were expressed as “ng·g^−1^ FW”. Total CK and IAA contents were analyzed with four biological replicates.

PA levels in sorghum mesocotyls were measured through HPLC. Samples (10 μL) were added in the reverse-phase (C18) column (6.0 mm × 150 mm; particle size, 5 mm; Shim-Pack CLC-ODS), followed by elution using 64/36 (*v*/*v*) methanol/water at a 0.8 mL·min^−1^ flow rate.

### 2.4. Assay of Starch, Phytohormones, Cell Morphogenesis-Related Enzyme Activity

The activities of α-amylose, β-amylose, α-glucosidase, 1-amicocyclopropane-1-carboxillic-acid synthase (ACS), acyl acid-amido synthetase (AAS), tryptophan aminotransferase (TAA), spermidine synthase (SPMS), spermidine synthase (SPDS), polyamine oxidase (PAO), β-xylosidase, endo-1,4-β-xylanas, and endo-β-mannanase were analyzed with corresponding enzyme-linked immune kits (Mlbio, Shanghai, China). Spectrophotometry was performed to determine color change with the enzyme mark instrument at 450 nm. Enzyme activities were analyzed by comparing sample ODs with standard curves.

### 2.5. Soluble Sugar and Glucose Content Analysis

The total soluble sugar content in sorghum seeds was determined with anthrone-H_2_SO_4_ colorimetry [34]. Briefly, seed samples (0.5 g) were dissolved in 10 mL distilled water. Thereafter, the mixed sample was added to a 30 mL test tube, extracted with a 100 °C water bath for a 30 min period, and diluted with distilled water to 25 mL. Later, the soluble sugar level was analyzed by comparing sample absorbance at 620 nm with a standard curve.

The glucose level in sorghum seeds was analyzed through HPLC according to the method of Li et al. (2022) [35]. Briefly, glucose extraction (10 μL) was added to the Spherisorb-NH_2_ column (Thermo Separation Products, Toulouse, France), followed by elution using 75/25 (*v*/*v*) acetonitrile/H_2_O_2_ with a Spectra Physics 8700 pump at the 0.8 mL·min^−1^ flow rate. The glucose level was examined by comparing the sample peak area with standard curves.

### 2.6. ATP and Energy Charge Analysis

HPLC was conducted to determine ATP and energy charge using the method of Liu et al. (2006) [36]. ATP content in sorghum seeds was expressed as “nmol·g^−1^ FW”. ATP content and energy charge were analyzed with four biological replicates.

### 2.7. Morphology Observation of Sorghum Mesocotyl

The cytomorphology of sorghum mesocotyl was observed with hematoxylin–eosin staining, according to Li et al. (2011) [37]. The middle section of sorghum mesocotyl germinated for 3 days was sampled and longitudinally cut for morphology observation.

### 2.8. Quantitative RT-PCR of Metabolism-Related Genes

Total RNAs of each sample were extracted and prepared to cDNA with PrimeScript™ RT reagent Kit (Takara, Dalian, China) through reverse transcription. LightCycler 480 Real-Time PCR system (Roche) was adopted for qPCR with SYBR-Green PCR Master kit (Applied Biosystems, Foster City, CA, USA). Supplementary Table S1 presents primers applied in the present work, with 18srRNA being internal control. FC = EΔCt was adopted to determine fold change of expression (FC), in which E represents the mean of amplification efficiency for one specific gene, while ΔCt stands for the difference in mean Ct among every biological replicate for 2 compared samples. Real-time PCR was conducted using 3 biological replicates, and 3 technical replicates were set for every biological replicate.

### 2.9. Statistical Analysis

The data were subjected to analysis of variance (ANOVA) to detect statistical differences using the SAS (8.0) statistics program. The multiple comparison for mean values were performed by Tukey’s honestly significant difference (HSD) test (*p* < 0.05). Percentage data were converted (before statistical comparison) by y = arcsin[sqrt (x/100)].

## 3. Results

### 3.1. Dynamic Changes of Seedlings at Deep-Sowing Condition

**Figure 1:**
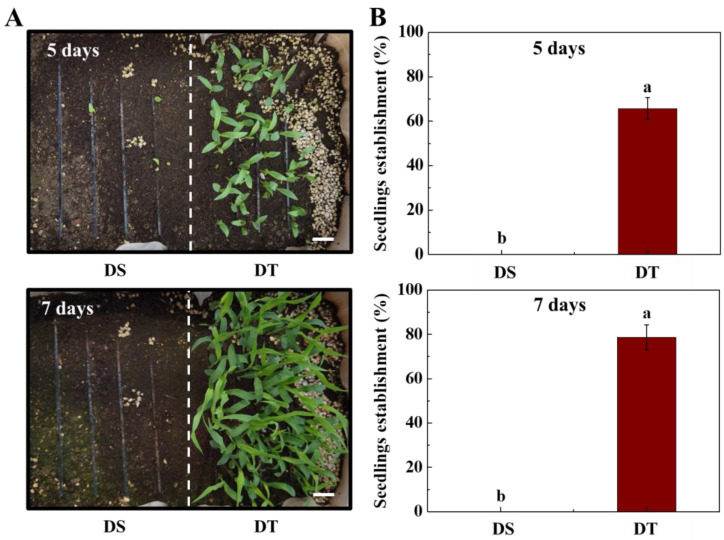
Deep-sowing emergence of sorghum seeds. (**A**) The deep-sowing emergence phenotype of sorghum seeds at 5 and 7 days of germination time. Scale bar, 3.0 cm. (**B**) Seedling emergence rate. DS: deep-sowing-sensitive material; DT: deep-sowing-tolerant sorghum. Sorghum seeds were sowed in the soil at 15 cm under a light/dark cycle of 12 h/12 h at 25 °C. The lowercase letter(s) on top of the bars indicate significant differences (*p* < 0.05, Tukey) across treatments.

**Figure 2:**
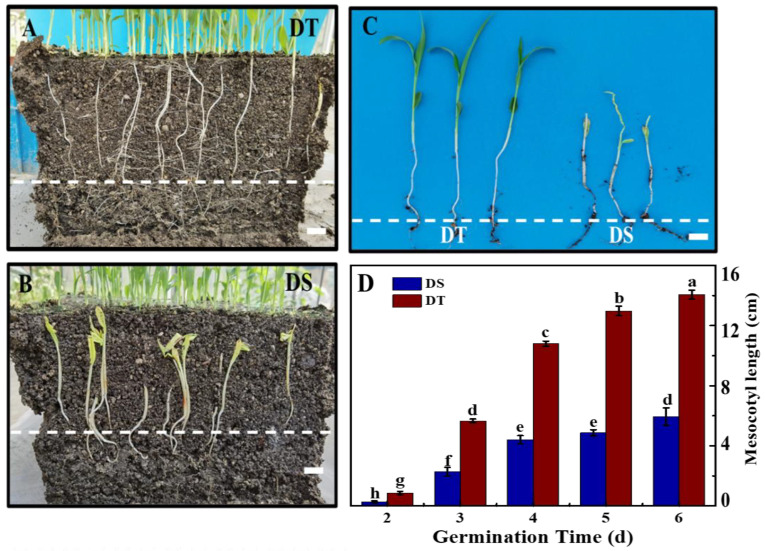
Seedlings characteristics of sorghum seeds in deep-sowing condition. (**A**) The underground seedling phenotype of DT seeds. Scale bar, 2.5 cm. (**B**) The underground seedling phenotype of DS seeds. Scale bar, 2.5 cm. (**C**) The seedling phenotype of DT and DS seeds. Scale bar, 3.5 cm. (**D**) The mesocotyl length of sorghum seedlings. DS: deep-sowing-sensitive material; DT: deep-sowing-tolerant sorghum. Sorghum seeds were sowed in 15 cm soil under a light/dark cycle of 12 h/12 h at 25 °C for 7 days. The dotted line represents the horizontal position of sorghum seeds. The lowercase letter on top of the bars in D is indicative of significant differences (*p* < 0.05, Tukey) across treatments.

In order to explore the potential mechanisms underlying the deep-sowing tolerance differences between DT and DS, we first observed the seedling establishment at deep-sowing conditions (Figure 1). No significant difference in seedling establishment was detected between DT and DS seed sowing at 5 cm for 7 days (Figure S1). However, under the deep-sowing condition, the seedling establishments of DT at 5 and 7 days of germination were 67.8% and 78.8%, respectively, while no DS seeds succeeded in establishing seeding. The seedling characteristic analysis showed no significant difference in coleoptile length between two sorghum lines at 7 days of germination (Figure S2). However, the mesocotyl length of DT was significantly longer than that of DS. At 7 days of germination, the mesocotyl length of DT reached 14.1 cm, while the mesocotyl length of DS was only 5.9 cm (Figure 2). Accordingly, it was speculated that the difference in the deep-sowing-sensitivity of the two lines was mainly regulated by the mesocotyl elongation.

### 3.2. Dynamic Changes of Starch Degradation in Sorghum Seeds at Deep-Sowing Condition

**Figure 3:**
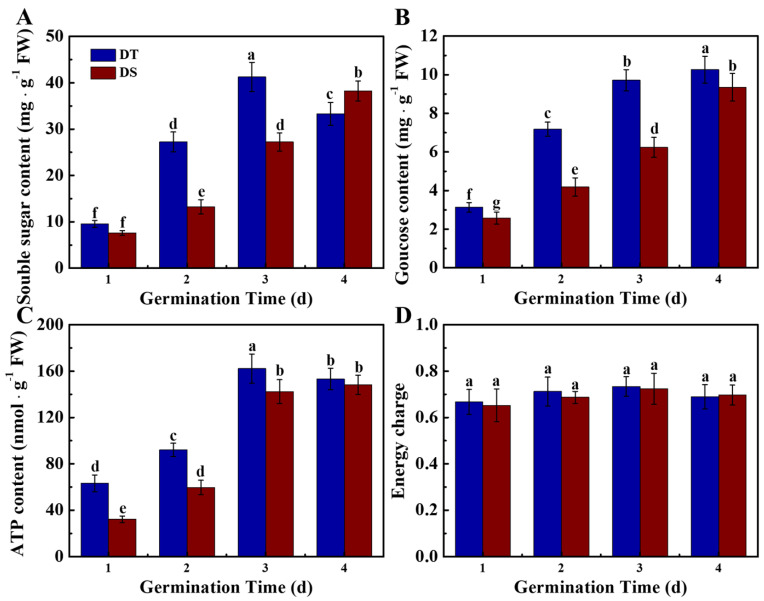
The contents of soluble sugar (**A**), glucose (**B**), ATP (**C**), and energy charge (**D**) in sorghum seeds during germination in deep-sowing conditions. DS: deep-sowing-sensitive material; DT: deep-sowing-tolerant sorghum. Sorghum seeds were sowed in 15 cm soil under a light/dark cycle of 12 h/12 h at 25 °C. The diverse lowercase letter(s) on top of the bars are indicative of significant differences (*p* < 0.05, Tukey) across treatments.

Seed storage substances were the major energy sources for seed germination and seedling emergence. During sorghum seed germination, glucose was the main nutrient produced through starch degradation. To further analyze the DST difference mechanism in DS versus DT seeds, glucose, soluble sugar, energy charge, and ATP levels within sorghum seeds were measured in the germination stage (1–4 d) (Figure 3). The soluble sugar and glucose contents in DS seeds were significantly lower than those in DT seeds at 2 and 3 days of germination. Moreover, significantly increased ATP contents were observed in DT seeds compared with DS seeds, while no significant difference in energy charge was detected between the two sorghum lines. The above findings revealed that a better energy supply may be an important cause for the deep-sowing-tolerance of DT seeds.

**Figure 4:**
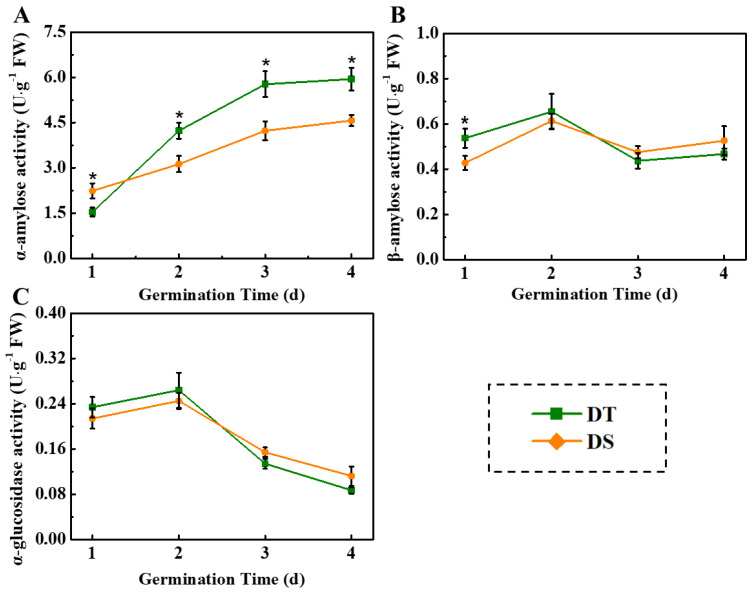
The activities of α-amylose (**A**), β-amylose (**B**), and α-glucosidase (**C**) in sorghum seeds during germination in deep-sowing conditions. DS: deep-sowing-sensitive material; DT: deep-sowing-tolerant sorghum. Sorghum seeds were sowed in 15 cm soil under a light/dark cycle of 12 h/12 h at 25 °C. The asterisk (*) indicates significant differences (*p* < 0.05, Tukey) across treatments at the same time point.

There was no significant difference in starch content between DT and DS seeds, while DT seeds contained a higher level of amylose and a lower level of amylopectin. The dynamic curve of the amylolysis rate in DS seeds displayed the delayed effect relative to DT (Figure S3). The enzyme analysis demonstrated that the α-amylose activity was gradually enhanced during the sorghum seed germination (Figure 4). The α-amylose activity in DT seeds was significantly lower than that in DS seeds on day 1 of germination; however, it rapidly increased on days 2–4 of germination, becoming significantly higher than that in DS seeds. The higher β-amylose activity was observed only in DT seeds on day 1 of germination. The α-glucosidase activity was maintained at a high level on days 1 and 2 of germination, which substantially declined on days 3 and 4 of germination, and no difference was observed in α-glucosidase activity between two sorghum lines. Consistent with enzyme activity analysis, the transcript levels of *Amy1* and *Amy2* in DT seeds were significantly higher than those in DS seeds (Figure S4).

### 3.3. Dynamic Changes of Phytohormones in Sorghum Mesocotyl Under the Deep Seeding Condition

**Figure 5:**
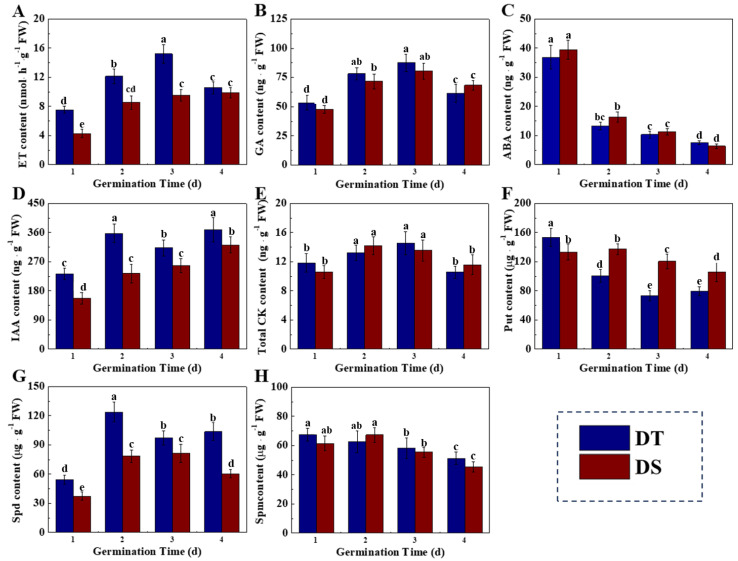
The contents of ET (**A**), GA (**B**), ABA (**C**), IAA (**D**), total CK (**E**), Put (**F**), Spd (**G**), and Spm (**H**) in sorghum mesocotyl during germination in deep-sowing condition. DS: deep-sowing-sensitive material; DT: deep-sowing-tolerant sorghum. ET: ethylene; GA: gibberellin; ABA: abscisic acid; IAA: indole-3-acetic acid; total CK: total cytokinin; Put: putrescine; Spd: spermidine; Spm: spermine. Sorghum seeds were sowed in 15 cm soil under a light/dark cycle of 12 h/12 h at 25 °C. The lowercase letter(s) on top of the bars indicate significant differences (*p* < 0.05, Tukey) across treatments.

Significant differences in the level of several phytohormones were detected between DT and DS during deep-sowing germination (Figure 5). The ET content in DT at 1, 2, and 3 days of germination was significantly higher than those in DS. In addition, significantly increased IAA content was observed in DT seeds compared with DS seeds. Meanwhile, no significant differences in ABA, GA, and total CK content were detected between the two sorghum lines. The Put content in DT was significantly lower than DS at 2–4 days of germination. On the contrary, the Spd content was up-regulated in DT compared with DS.

**Figure 6:**
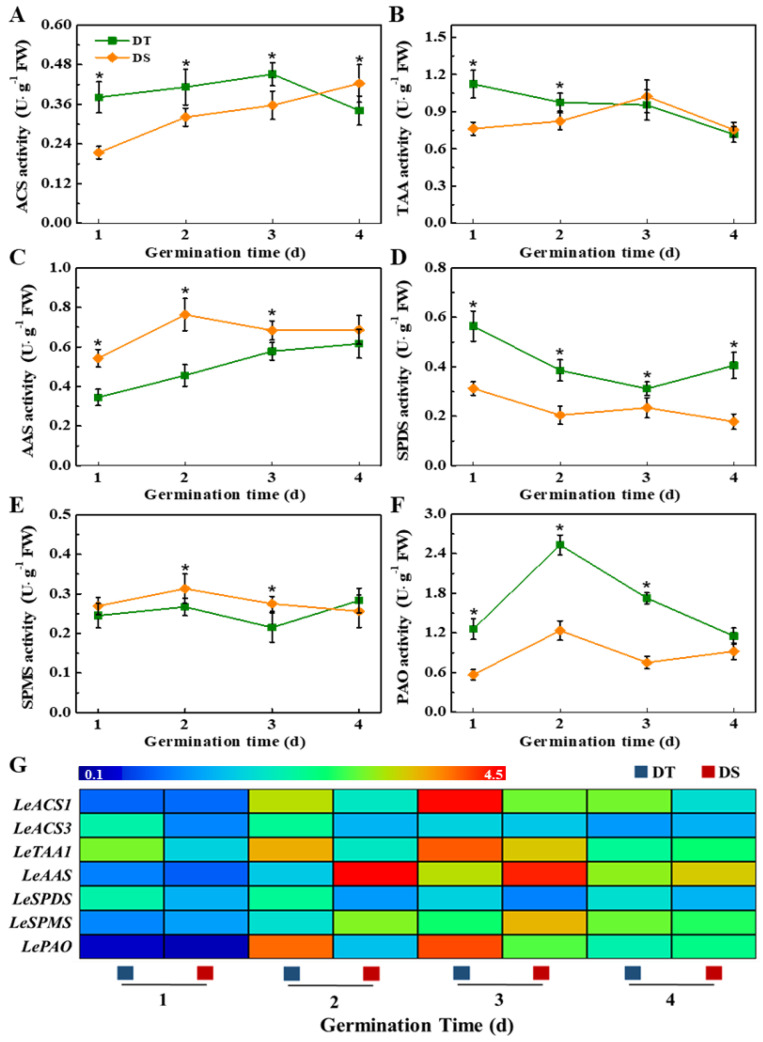
The activities of ACS (**A**), TAA (**B**), AAS (**C**), SPDS (**D**), SPMS (**E**), PAO (**F**), and transcriptional levels of corresponding genes (**G**) in sorghum mesocotyl during germination at deep-sowing condition. DS: deep-sowing-sensitive material; DT: deep-sowing-tolerant sorghum. ACS: 1-amicocyclopropane-1-carboxillic-acid synthase; TAA: tryptophan aminotransferase; AAS: acyl acid-amido synthetase; SPDS: spermidine synthase; SPMS: spermine synthase; PAO: polyamine oxidase. Sorghum seeds were sowed in 15 cm soil under a light/dark cycle of 12 h/12 h at 25 °C. The asterisk (*) indicates significant differences (*p* < 0.05, Tukey) across treatments at the same time point. Real-time quantitative PCR was performed using three biological replications, and each was made in three technical replicates. The Illustrator software was used for creating the heat map [38].

The results of enzyme analysis were consistent with the dynamic phytohormone changes (Figure 6A–F). DT showed remarkably higher 1-amicocyclopropane-1-carboxillic-acid synthase (ACS) activity at 1–3 days of germination. Tryptophan aminotransferase (TAA) and acyl acid-amido synthetase (AAS) are the key enzymes responsible for IAA synthesis, which negatively and positively regulate IAA synthesis, respectively. On days 1 and 2 of germination, the TAA activity in DT was significantly lower than that in DS, while the AAS activity was remarkably higher than in DS.

Additionally, the spermidine synthase (SPDS) and polyamine oxidase (PAO) exhibited higher activities during the germination process of DT, while the spermine synthase (SPMS) activity was higher in DS (Figure 6G). Moreover, significantly higher transcriptional levels of *LeACS1*, *LeACS3*, *LeTAA1*, *LeSPDS*, and *LePAO* were detected in DT seeds compared with DS seeds. Meanwhile, the expressions of *LeAAS* and *LeSPMS* in DT seeds were remarkably lower than those in DS seeds. Moreover, it was worth noting that the content of H_2_O_2_, the product of Spd oxidation catalyzed by PAO, was highly accumulated in the DT mesocotyl (Figure S5). The above findings indicated that the differences in the activities of phytohormone-related enzymes and the corresponding gene expressions lead to differences in phytohormone contents during the deep-sowing germination of two sorghum lines. The metabolism of ET, IAA, and polyamine may play an important role in the responses of these two sorghum lines to the sensitivity of deep-sowing-tolerant seedlings.

### 3.4. Cytological Changes of Mesocotyl Under the Deep-Sowing Condition

**Figure 7:**
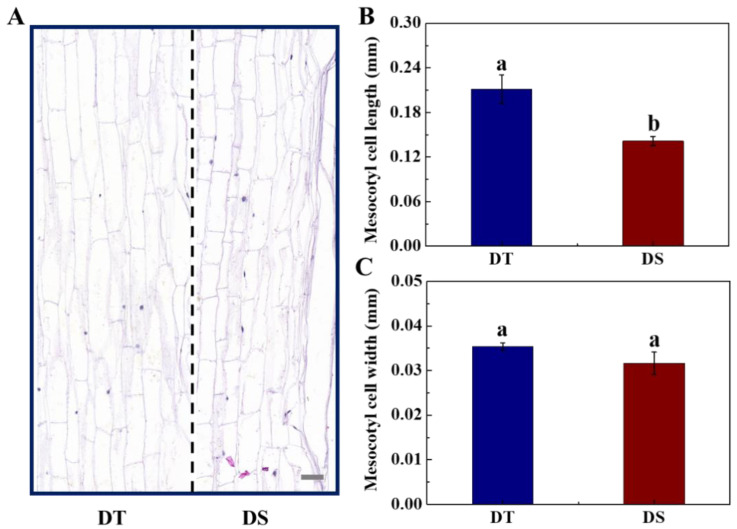
The cell morphology (**A**), cell length (**B**), and cell width (**C**) in sorghum mesocotyl during seed germination in deep-sowing condition. DS: deep-sowing-sensitive material; DT: deep-sowing-tolerant sorghum. Sorghum seeds were sowed in soil at 15 cm under a light/dark cycle of 12 h/12 h at 25 °C for 5 days. The middle mesocotyl tissue was used for cell morphology observation. Scale bar, 0.04 mm. The lowercase letter(s) on top of the bars indicates significant differences (*p* < 0.05, Tukey) across treatments.

During the deep-sowing germination, the division and expansion of mesocotyl cells are vital for mesocotyl elongation. Therefore, in the present study, we further detected the mesocotyl cell morphology of sorghum (Figure 7). The results showed that the mesocotyl cell length in DT was significantly higher than in DS, while no significant difference in cell width was observed between the two lines. After 5 days of germination, the longitudinal mesocotyl cell number in DT was significantly higher than that in DS. The above results show that cell division and cell expansion jointly regulated the mesocotyl elongation of sorghum and affected the emergence ability of sorghum seeds under the deep-sowing condition.

**Figure 8:**
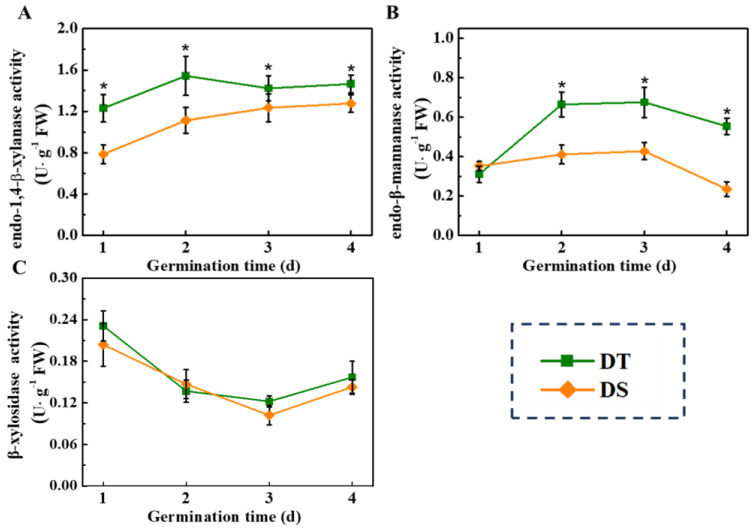
The activities of endo-1,4-β-xylanase (**A**), endo-β-mannanase (**B**), and β-xylosidase (**C**) in sorghum mesocotyl during germination at deep-sowing condition. DS: deep-sowing-sensitive material; DT: deep-sowing-tolerant sorghum. Sorghum seeds were sowed in 15 cm soil under a light/dark cycle of 12 h/12 h at 25 °C. The asterisk (*) indicates significant differences (*p* < 0.05, Tukey) across treatments at the same time point.

Furthermore, the activities of several key cell wall metabolic-related enzymes and the corresponding gene expressions were measured in the present study (Figure 8). Consistently, the activities of endo-1,4-β-xylanase and endo-β-mannanase in the DT mesocotyl were significantly higher than those in DS. Moreover, significantly higher transcript levels of *LeMAN* and *LeMYLA* were observed in DT mesocotyl at 1 and 2 days of germination (Figure S6), while the β-xylosidase activity did not show any significant difference between the two lines. It has been suggested that cell wall weakening is closely involved in mesocotyl elongation under deep-sowing conditions.

## 4. Discussion

Under deep-sowing conditions, seedling emergence depends on mesocotyl elongation rather than on coleoptile elongation [39,40,41]. Likewise, according to our results, the mesocotyl length of DT was significantly higher than that in DS, and no significant difference in coleoptile length was detected between the two sorghum lines. Mesocotyl is the major factor that affects germination dynamics in monocotyledonous crops, and it is widely utilized as an experimental system for investigating the deep-sowing mechanism. Nonetheless, few studies have explored the mesocotyl elongation and deep-sowing resistance mechanisms in sorghum seeds. Our results indicated that mesocotyl elongation ability was related to the sorghum seedling emergence from deep soil. Phytohormones and energy metabolism contributed to the difference in mesocotyl elongation, hence, seedling emergence between DT and DS sorghum varieties.

The roles of phytohormones, mainly GA, ABA, IAA, ETH, and CK, in controlling the mesocotyl growth of seeds have been widely reported in rice. Cao et al. (2005) reported that exogenous GA, CK, and IAA within optimum concentration promoted mesocotyl elongation in rice, among which GA achieved the most obvious effect, followed by CK and IAA [42]. CK can induce cell division to promote mesocotyl growth, while GA and IAA promote mesocotyl cell elongation [19]. The overlying depth and compactness of soil can affect the synthesis of endogenous ETH in *Arabidopsis thaliana* and rice, thus contributing to seed germination [40,43]. In the present study, we found that the mesocotyl of DT possessed higher contents of ET and IAA, and there was no significant difference in ABA, GA, or total CK content between the two lines. Further enzyme activity analysis and gene expression detection suggested that the higher ET content in the mesocotyl of DT was induced by the high expression of *LeACS1* and *LeACS3* and the high activity of ACS, while the accumulation of IAA was closely associated with the activities of TAA and AAS and the corresponding gene expressions. The above results demonstrate that ET and IAA may exert a vital role in the mesocotyl elongation of sorghum seeds under the deep-seeding germination condition. The role of phytohormones in regulating mesocotyl elongation may vary in different crops. Polyamines (PAs), the small aliphatic polycationic nitrogenous compounds, mainly include putrescine (Put), spermine (Spm), and spermidine (Spd) [44]. Among which, Spd is the major triamine that has been suggested to be important for regulating plant growth and development, including seed germination, flower differentiation, and fruit formation and senescence [45,46,47]. The present study also showed a significant difference in polyamine metabolism in the mesocotyls between DT and DS. Compared with DS, the mesocotyl in DT had a higher Spd content and a lower Put content, accompanied by massive H_2_O_2_ accumulation. Furthermore, the SPDS and PAO activities and corresponding gene expressions in DT were markedly higher than those in DS. SPDS mainly functions to catalyze the conversion of Put into Spd, while PAO is mainly responsible for the oxidation of SPD and SPM and the production of H_2_O_2_ [44,48]. We speculated that the excessive H_2_O_2_ accumulation in the mesocotyl of DT might be closely related to the SPD synthetic and oxidative pathways. Similarly, a previous study indicated that polyamine metabolism and the induced H_2_O_2_ accumulation positively regulate mesocotyl cell division and elongation in maize [49].

Starch degradation into soluble sugars exerts an important effect on promoting seed germination and early seedling emergence. There are two enzymic routes involved in degrading starch within cereals, including amylase-catalyzed hydrolysis and starch phosphorylase-catalyzed phospholysis. Compared with β-amylose, α-glucosidase and α-amylase were tightly linked to seed germination of maize and rice [28]. The present study found that the α-amylose activity increased rapidly during sorghum seed germination, while the α-amylose activity of DT was significantly higher than that of DS. There was no significant difference in β-amylose and α-glucosidase activity between the two lines. Consistently, significantly higher levels of soluble sugar, glucose, and ATP were detected in DT seeds during deep-sowing germination. α-amylase is usually detected within the aleurone layer and scutellar epithelial cells of germinating cereal seeds [50,51]. α-amylase is produced by the aleurone layer and released into the endosperm to catalyze hydrolysis of the stored starch to maltose and maltotriose, which were then transformed into glucose under the action of a-glucosidase [52]. The ATP obtained from glycolysis has an essential effect on supporting seed germination and seedling emergence [51]. It was proposed that starch catabolism and energy supply might be tightly associated with the deep-sowing resistance of sorghum seeds. Additionally, we also found that the starch composition was different between the two lines of seeds. DT seeds showed higher amylose/amylopectin proportions, which might be the potential cause of the higher amylolysis rate in DT.

Cell division and expansion are the basis of mesocotyl elongation [11]. From the perspective of cell morphology, cell elongation in the rice mesocotyl makes a greater contribution to mesocotyl elongation than cell division [53]. The present study found that cell number and cell length in DT mesocotyl were remarkably higher than that in DS. It was speculated that mesocotyl elongation during the deep-sowing germination of sorghum seeds was jointly regulated by cell division and cell expansion. The cell wall exerts a vital impact on cell elongation, expansion, and morphogenesis, and the weakened cell wall is beneficial for mesocotyl elongation [24]. The cell wall mainly consists of the primary cell wall, secondary cell wall, and the intercellular layer, among which the primary cell wall has a greater hardness [54]. It is reported that increases in cell diameter and longitudinal elongation are mainly restricted by the secondary cell wall. Enzymes that can degrade hemicellulose in the secondary cell wall of mesocotyl cells mainly include xylanase and mannanase [55]. Our study revealed that mesocotyl in DT showed higher activities of endo-1,4-β-xylanase and endo-β-mannanase, as well as increased corresponding gene expressions. Consistently, it was reported that the endo-β-mannanase activity increased rapidly during the seed germination in *Arabidopsis thaliana* and tomato [56,57]. (Iglesias-Fernández et al., 2010; Chen et al., 2018), and the activities of endo-1,4-β-xylanase and endo-β-mannanase increased to varying degrees in the case of Chinese cabbage and rice [58,59].

## 5. Conclusions

The present study demonstrated that mesocotyl elongation is related to the process of seedling emergence under the deep-seeding condition in sorghum. The metabolism of phytohormones (mainly including IAA, ET, and PAs) and cell morphogenesis in mesocotyl might be involved in the mesocotyl elongation during deep-sowing germination. Additionally, higher starch decomposition activity and better energy supply in DT endosperm might be a reason for the high deep-sowing tolerance of DT. This work provides important insights into the molecular network of sorghum seed germination and seedling establishment in deep soil planting.

## Data Availability

The original contributions presented in this study are included in the article. Further inquiries can be directed to the corresponding author(s).

## Supplementary Materials

The following supporting information can be downloaded at https://www.mdpi.com/article/10.3390/plants14091366/s1, Figure S1. The seedling establishment of sorghum seeds sowing at 5 cm for 7 days. DS: deep-sowing-sensitive material; DT: deep-sowing-tolerant sorghum. The lowercase letter(s) on top of the bars indicate significant differences (*p* < 0.05, Tukey) across treatments. Figure S2. Coleoptile length of sorghum seedlings. DS: deep-sowing-sensitive material; DT: deep-sowing-tolerant sorghum. Sorghum seeds were sowed in 15 cm soil under a light/dark cycle of 12 h/12 h at 25 °C for 7 days. The lowercase letter(s) on top of the bars indicate significant differences (*p* < 0.05, Tukey) across treatments. Figure S3. The starch content (A) and degradation rate of starch (B), amylose (C), and amylopectin in sorghum seed during germination in the deep-sowing condition. DS: deep-sowing-sensitive material; DT: deep-sowing-tolerant sorghum. Sorghum seeds were sowed in soil at 15 cm under a light/dark cycle of 12 h/12 h at 25 °C. The diverse lowercase letter(s) on top of the bars indicate significant differences (*p* < 0.05, Tukey) across treatments. Figure S4. The gene expression of Amy1 (A) and Amy2 (B) in sorghum seed during germination in the deep-sowing condition. DS: deep-sowing-sensitive material; DT: deep-sowing-tolerant sorghum. Sorghum seeds were sowed in 15 cm soil under a light/dark cycle of 12 h/12 h at 25 °C. The diverse lowercase letter(s) indicates a significant difference (*p* < 0.05, Tukey) across treatments. Figure S5. The H_2_O_2_ content in sorghum mesocotyl during germination at deep-sowing condition. DS: deep-sowing-sensitive material; DT: deep-sowing-tolerant sorghum. Sorghum seeds were sowed in soil at 15 cm under a light/dark cycle of 12 h/12 h at 25 °C. The diverse lowercase letter(s) indicates significant differences (*p* < 0.05, Tukey) across treatments at the same time point. Figure S6. The gene expression of LeMAN (A) and LeMYLA (B) in sorghum mesocotyl during germination at deep-sowing conditions. DS: deep-sowing-sensitive material; DT: deep-sowing-tolerant sorghum. MAN: endo-β-mannanase; MYLA: endo-1,4-β-xylanase; Sorghum seeds were sowed in 15 cm soil under a light/dark cycle of 12 h/12 h at 25 °C. The diverse lowercase letter(s) indicates significant differences (*p* < 0.05, Tukey) across treatments. Table S1. Primers used in qRT-PCR analysis of genes expression.
