# Supplementary material for: Morphological, Biochemical, and Cytological Analyses of Deep-Sowing Tolerance in Sorghum Seeds
Source: Plants (Basel). 2025 Apr 30;14(9):1366. doi: 10.3390/plants14091366 (PMC12073692; doi:10.3390/plants14091366)
Supplement: Supplementary file 1 [file plants-14-01366-s001.zip › plants-3362546-supplementary.pdf]

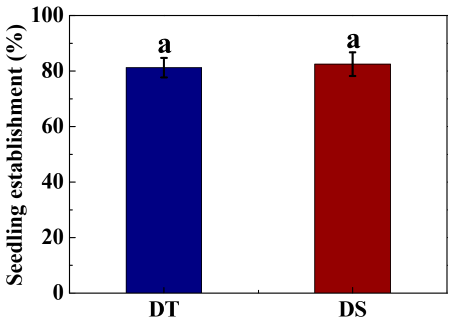

Supplementary Figure S1. The seedlings establishment of sorghum seeds sowing at 5 cm for 7 days. DS: deep-sowing sensitive material; DT: deep-sowing tolerant sorghum. The diverse lowercase(s) on top of the bars were indicative of significant differences ($p < 0.05$, Tukey) across treatments.

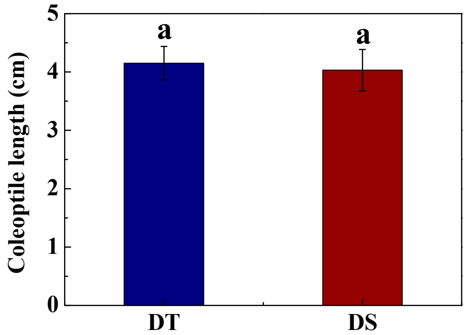

Supplementary Figure S2. Coleoptile length of sorghum seedlings. DS: deep-sowing sensitive material; DT: deep-sowing tolerant sorghum. Sorghum seeds were sowed in soil at 15 cm under a light/dark cycle of 12-h/12-h at 25 °C for 7 days. The diverse lowercase(s) on top of the bars were indicative of significant differences ($p < 0.05$, Tukey) across treatments.

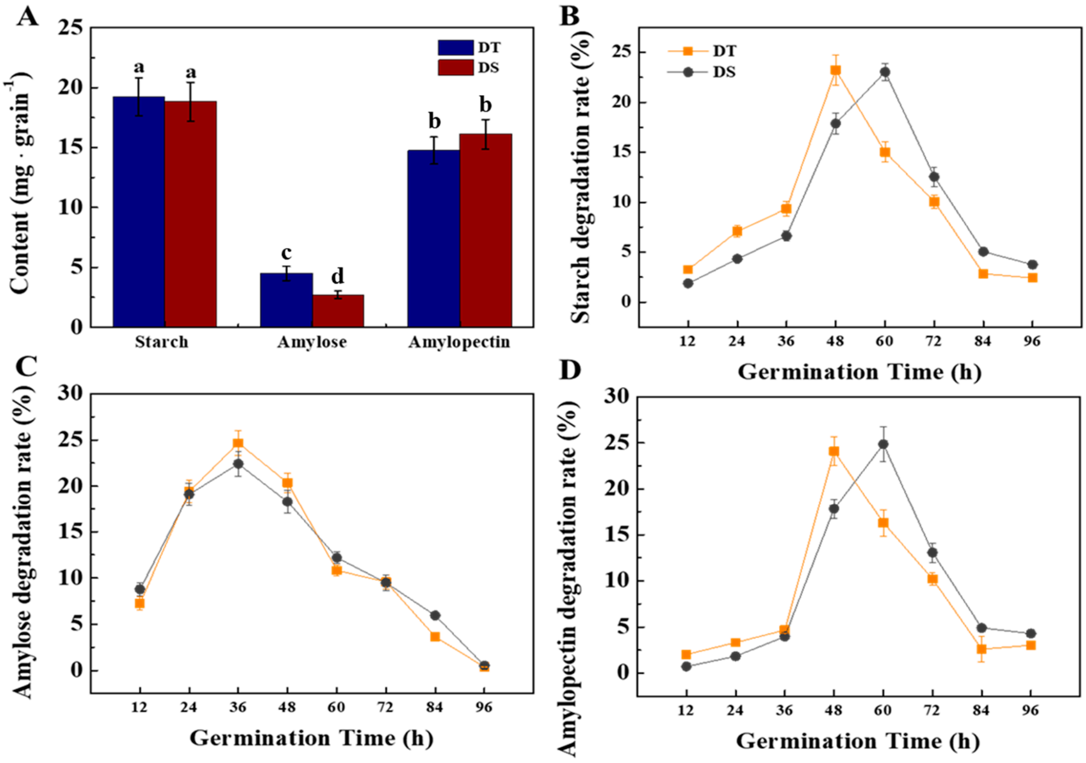

Supplementary Figure S3. The starch content (A) and degradation rate of starch (B), amylose (C) and amylopection in sorghum seed during germination at deep sowing condition. DS: deep-sowing sensitive material; DT: deep-sowing tolerant sorghum. Sorghum seeds were sowed in soil at 15 cm under a light/dark cycle of 12-h/12-h at 25 °C. The diverse lowercase(s) on top of the bars were indicative of significant differences (p < 0.05, Tukey) across treatments.

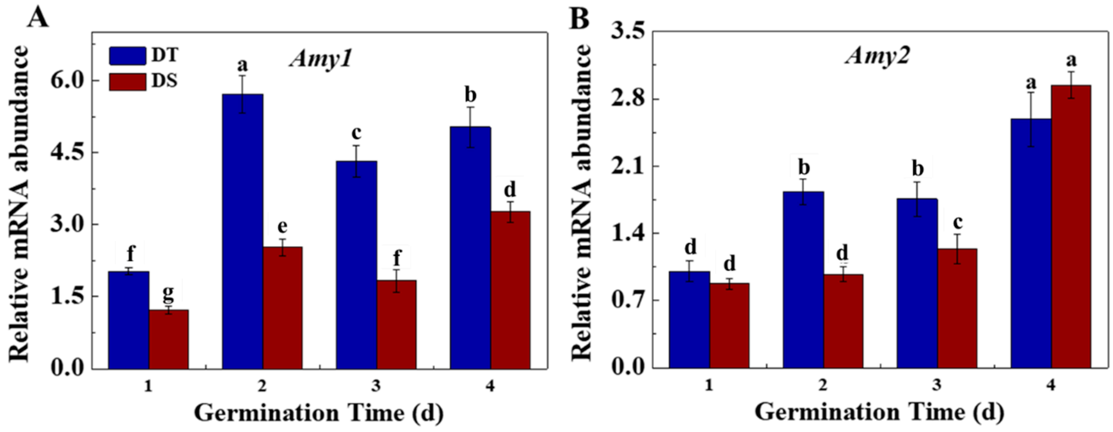

Supplementary Figure S4. The gene expression of *Amy1* (A) and *Amy2* (B) in sorghum seed during germination at deep sowing condition. DS: deep-sowing sensitive material; DT: deep-sowing tolerant sorghum. Sorghum seeds were sowed in soil at 15 cm under a light/dark cycle of 12-h/12-h at 25 °C. The diverse lowercase(s) indicates significant differences (p < 0.05, Tukey) across treatments.

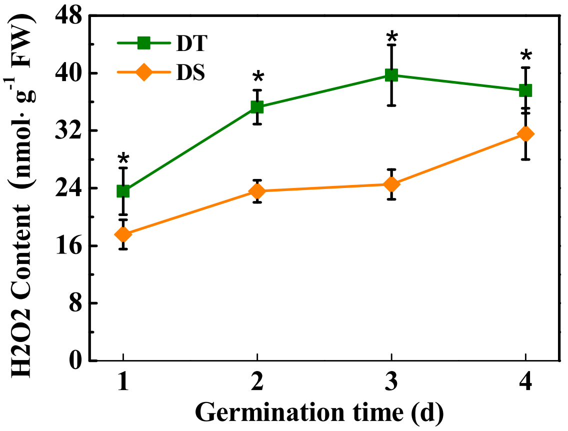

Supplementary Figure S5. The H_2O_2 content in sorghum mesocotyl during germination at deep sowing condition. DS: deep-sowing sensitive material; DT: deep-sowing tolerant sorghum. Sorghum seeds were sowed in soil at 15 cm under a light/dark cycle of 12-h/12-h at 25 °C. The diverse lowercase(s) indicates significant differences ($p < 0.05$, Tukey) across treatments at the same time point.

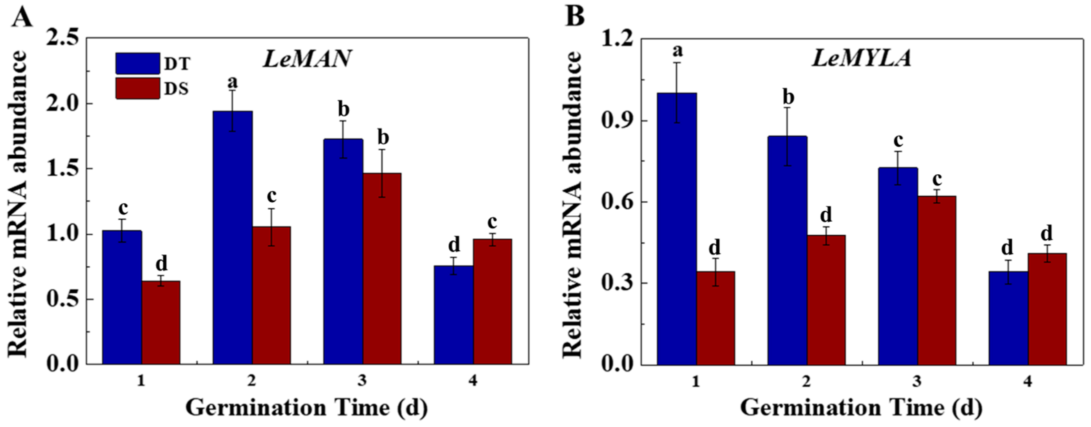

Supplementary Figure S6. The gene expression of *LeMAN* (A) and *LeMYLA* (B) in sorghum mesocotyl during germination at deep sowing condition. DS: deep-sowing sensitive material; DT: deep-sowing tolerant sorghum. MAN: endo-β-mannanase; MYLA: endo-1,4-β-xylanase; Sorghum seeds were sowed in soil at 15 cm under a light/dark cycle of 12-h/12-h at 25 °C. The diverse lowercase(s) indicates significant differences ($p < 0.05$, Tukey) across treatments.

Supplemental Table S1 Primers used in qRT-PCR analysis of genes expression

Gene name	Primer sequence	
	Forward primer	Reverse primer
18srRNA	CCGTGGTGGCCAGTAAGTTC	GGACTCAACATGGGCTCTGC
LeACS1	GCTCATCACCAACCCTTCCA	GCCGGAGTATATCTCGTCGC
LeACS3	GAGAAGATTAGGGGCGGCAA	TGAACGTGAGCAGCTCGTTA
LeTAA1	CCACAAGATACTGACGCGGA	TCACGAAGATGTCGTAGGCG
LeAAS	GCGCTCTACTTCCTGTTCGT	GTAGTAGCTCGTCAGCACGG
LeSPDS	CATTGGAGATGGTGTCGCCT	CACCGACTGGAAGAAAGGCT
LeSPMS	AGGACCACCGGTCAACTTTC	AAACGCTGCCCTATGCATCT
LePAO	CTCCAGCTCAACAAGGTGGT	TAGTCTGCCCTGTACACCGA
LeAmy1	TCAACTGGCTCAAGACGGAC	GCCAGCGAGGTCCATATCTC
LeAmy2	CAAGTGCAAGGTCGTTGTCG	TCTGAAGGAGGCTGATCCCA
LeMAM	GTGTGAATCCTGGGGACTGG	ATAAGCGTGAACCGAGGCAA
LeMYLA	CTGCGTCATGTGCTCCTACA	GAGACGATGTACCCGTGGAG
